# Assessing of Celiac Disease and Nonceliac Gluten Sensitivity

**DOI:** 10.1155/2015/723954

**Published:** 2015-04-29

**Authors:** N. Ontiveros, M. Y. Hardy, F. Cabrera-Chavez

**Affiliations:** ^1^Unidad Académica de Ciencias de la Nutrición, Universidad Autónoma de Sinaloa, Avenida Cedros y Calle Sauces, S/N, Fracc. Los Fresnos, 80019 Culiacán, SIN, Mexico; ^2^Programa Regional de Doctorado en Biotecnología, FCQB, Universidad Autónoma de Sinaloa, Ciudad Universitaria, 80040 Culiacán, SIN, Mexico; ^3^Immunology Division, Walter and Eliza Hall Institute of Medical Research, 1G Royal Parade, Parkville, VIC 3052, Australia

## Abstract

The publication of papers on the topic of gluten related disorders has substantially increased over the last few years. This has motivated healthcare professionals to pay attention not only to celiac disease and wheat allergy but also to a condition termed nonceliac gluten sensitivity (NCGS). Until now this condition has been diagnosed clinically on the basis of exclusion criteria and clinical response to gluten withdrawal. In addition, recent research in this field has shown that other food components distinct from gluten are implicated in NCGS cases, thereby changing our general understanding of NCGS diagnosis in either individuals on gluten containing diets or those already following a gluten-free diet with no proper diagnostic work-up of celiac disease. With this in mind, the assessment of NCGS will require extensive knowledge of celiac disease manifestations and the laboratory tests commonly performed during diagnosis of celiac disease.

## 1. Introduction

Celiac disease (CD) and nonceliac gluten sensitivity (NCGS) are thought to be two different clinical conditions triggered by the ingestion of wheat/gluten in susceptible individuals. The former condition is strongly associated with ingestion of oral gluten from wheat and other gluten sources such as rye and barley. NCGS has also been associated with the intake of gluten, but other components also found in wheat could be the triggers of the symptoms seen in NCGS cases [1–3]. Notably and different from CD, the biomarkers for the diagnostic work-up of NCGS remain unknown and the oral gluten related symptoms, such as gastrointestinal or neurological symptoms, are the hallmarks of this condition [4–6].

CD is a well-established T-cell-mediated autoimmune enteropathy with a strong genetic component and variable clinical manifestations (ranging from asymptomatic to global malabsorption) [7]. Human leukocyte antigen (HLA) haplotypes DR3-DQ2.5, DR5-DQ7/DR7-DQ2.2, and DR4-DQ8 are the main genetic risk factors associated with CD and the absence of their respective alleles practically excluded the condition. In contrast, NCGS is thought to be a condition where gluten related adverse reactions occur despite an absence of CD and other intestinal inflammatory disorders. Furthermore, NCGS is not recognized as a strict enteropathy and it is unclear whether gluten-associated symptoms can be transient in some patients.

The current treatment recommendation for CD patients is strict gluten-free diet with clinical follow-up due to the health complications such as nutritional deficiencies, malignancy, and autoimmune diseases that are more prevalent in untreated CD [8–12]. In comparison, NCGS is not thought to cause nutritional deficiencies or higher rates of malignancies [12]. In addition, evidence is mounting that NCGS patients do not require a life-long gluten-free diet and monitoring but they are better suited to other exclusion diets [1, 2].

Due to the lack of both biomarkers and an approved diagnostic approach to assess for NCGS, there have been proposed algorithms to differentiate between CD and NCGS [13, 14]. Currently, it is reasonable to assess NCGS based on the exclusion of other gluten related disorders and clinical response to restrictive diets. However, due to the wide spectrum of CD and those cases already following gluten-free diet without proper diagnostic work-up, this assessment would require not only extensive knowledge of CD manifestations in infants and adults, but also adequate interpretation of the CD-associated laboratory tests.

The aim of this review is to give an updated overview of the spectrum of CD in light of recently published definitions of gluten related disorders and to describe the clinical/laboratory characteristics of NCGS and the potential coexistence of NCGS with other gastrointestinal disorders. We also aimed to discuss the clinical utility of current tools for the diagnostic work-up of CD in order to rule in/out NCGS.

## 2. Clinical Manifestations of CD and Subtypes

CD manifestations in both children and adults may be difficult to recognize because of the variation in signs and symptoms associated with the condition. Most of the patients attending primary care and gastroenterology clinics present predominantly with gastrointestinal symptoms such as diarrhea, bloating, abdominal pain, and constipation [15, 16]. Common symptoms in children under 5 years old include diarrhea, distension, and abdominal pain [17]. However, the frequency of classical CD has dropped substantially, and more commonly cases are identified as nonclassical CD (Table 1) [18, 19]. Common extraintestinal symptoms include failure to thrive, weight loss, anemia, and short stature [16, 17]. In adults, a recent study carried out in an Iranian population showed that dyspepsia, diarrhea, anemia, and short stature were the most common complaints [20]. Other potential extraintestinal symptoms include weakness, lethargy, and headache. Thus, the coexistence of gastrointestinal and extraintestinal symptoms reinforces the clinical suspicion of CD.

The updated definition of CD provided by the current European Society for Paediatric Gastroenterology, Hepatology and Nutrition (ESPGHAN) guidelines implies three criteria that ideally should be fulfilled to diagnose the condition: CD-specific antibodies, genetic background, and enteropathy [21]. Furthermore, the presence/absence of CD-associated symptoms is useful for classification of the condition into different subtypes. It is critical to test these criteria in order to rule out CD before assessment for other wheat/gluten related disorders.

Several terms have been used interchangeably in the literature to define CD subtypes. This makes it difficult to correctly identify the terms that should be used to describe particular CD cases. To overcome this, a panel of CD experts proposed new definitions for CD subtypes and other wheat/gluten related disorders (the Oslo definitions for CD and related terms) [22]. The authors discourage the use of the terms asymptomatic, typical, atypical, overt, and silent CD and encourage the use of the terms classical, nonclassical, subclinical, and potential CD. Each term is defined in part on the basis of signs and symptoms of malabsorption.

Current ESPGHAN guidelines for CD diagnosis encourage the use of the terms silent, latent, and potential CD as well as the use of gastrointestinal and extraintestinal signs and symptoms [21]. According to these guidelines and the Oslo definitions for CD [22], the terms silent, asymptomatic, and subclinical CD could be used to denote those patients carrying the HLA haplotypes DR3-DQ2.5, DR5-DQ7/DR7-DQ2.2, and/or DR4-DQ8 with positive CD-specific antibodies and biopsy findings compatible with CD, but without signs or symptoms associated with the condition. These patients are often detected through CD screening programs or testing of high risk subjects such as those with type 1 diabetes mellitus, IgA nephropathy, and Williams syndrome [21].

Potential CD denotes patients carrying the CD-associated HLA haplotypes with positive CD-specific serology, but without histological abnormalities in small bowel biopsies [21, 22]. Table 1 shows a classification of CD subtypes compatible with current ESPGHAN guidelines and those proposed by the Oslo definitions for CD [21, 22]. The terms used cover the spectrum of CD manifestations and weight is given to the type of symptoms. According to the Oslo definitions for CD, at least five definitions of latent CD have been described in the literature, and as such this confusion has made it a subtype particularly difficult to diagnose [22]. Thus, use of the term latent CD is discouraged. As stated by Ferguson et al. [39], the term latent CD should only be applied to patients with normal jejunal biopsy while taking a normal diet, but at some other time they have had a flat jejunal biopsy which recovers on a gluten-free diet. Thus, “only rarely and usually by chance, for example, previous biopsy in a research investigation, does a patient fulfill criteria for latent coeliac disease” [39].

## 3. Clinical/Laboratory Characteristics and Coexistence of NCGS

According to Troncone and Jabri [40], the term gluten sensitivity (GS) is employed to describe conditions triggered by gluten without precise definitions and for which there is no knowledge of the underlying mechanisms. The Oslo definitions for CD encourage the use of the term gluten related disorders to define conditions associated with oral gluten [22]. Moreover, define NCGS as a condition in which oral gluten leads to morphological or symptomatic manifestations despite the absence of CD and other intestinal inflammatory disorders. NCGS has additionally been defined by Sapone et al. [13, 41] as those cases of gluten adverse reaction in which wheat allergy, CD, inflammatory bowel disease (IBD), type I diabetes, and* Helicobacter pylori* infection have been ruled out. Thus, current NCGS definitions and diagnoses are based on exclusion criteria. However, to obtain a confirmed diagnosis of NCGS, a double blind gluten-placebo-controlled test would be required.

These definitions of NCGS are restricted to gluten; however other food components could trigger some of the symptoms associated with NCGS. For instance, the allergenic wheat component *α*-amylase inhibitor 0.19 [3, 42] and FODMAPs (fermentable oligosaccharides, disaccharides, monosaccharides, and polyols) could contribute to this condition [1, 2]. If these observations were taken into account, it would be difficult to assess the gluten-specific symptoms associated with NCGS separately from symptoms due to other dietary components in daily clinical practice. This also highlights that NCGS, if it does exist, is a complex disorder. In general, we agree with views of Gibson and colleagues that future studies on NCGS should rule out CD by HLA typing and/or histological and immunological criteria (including intraepithelial lymphocytosis) and that credence should be given to other wheat-related food constituents besides gluten as a trigger for gastrointestinal symptoms [43]. Furthermore, wheat allergy should be ruled out on the basis of objective diagnostic criteria [12, 13]. In fact, except for those cases where the cause of a severe food allergy reaction can be clearly identified, food allergy diagnosis should be confirmed by food challenge test, ideally double-blinded and placebo-controlled [44].

NCGS and CD cannot be distinguished clinically due to symptoms, either intestinal or extraintestinal, which largely overlap between the two conditions. Furthermore, in both conditions symptomatic relief is reached after gluten withdrawal, the latter of which is also seen in IgE mediated wheat allergy. Certainly, some classical symptoms of wheat allergy reaction could differ from those seen in NCGS and CD, that is, cough and wheezing, urticaria or erythema, and trouble breathing, but gastrointestinal symptoms are also common [42]. Thus, the laboratory characteristics of those patients suspected of NCGS are of particular relevance in clinical practice. NCGS patients present negative CD-specific serology, may or may not carry HLA genes compatible with CD, and do not present the gluten induced intestinal damage characteristic of CD [5, 12–14, 22, 40, 45] (Table 1). Furthermore, wheat allergy tests have to be negative over time given the fact that delayed allergic reactions may occur when undertaking oral wheat challenge tests [42]. Unfortunately, there are no international consensus statements on diagnosing delayed wheat/food-related symptoms, and those that appear between 2 hours and up to five days following the oral challenge are commonly considered as delayed [42, 46].

It has been proposed that intestinal inflammatory conditions such as Crohn's disease and ulcerative colitis should also be ruled out before diagnosing NCGS [22, 41]. However, Vojdani and Perlmutter [47] reported a case of NCGS with overlapping Crohn's disease. The case is well documented with regard to symptoms, laboratory examinations, and clinical interventions. CD was ruled out by negative CD-specific serology and histology while the patient was on a gluten containing diet. However, wheat allergy was not ruled out and the potential effect of other nongluten wheat components was not controlled. We believe that until specific biomarkers for the diagnostic work-up of NCGS become available and a well documented definition of NCGS is given, the coexistence of inflammatory bowel disease (IBD) and NCGS can be a subject for debate. Indeed, there are several reports supporting that CD and IBD can coexist [48–53], and it has been shown that the prevalence of IBD in CD patients could be up to tenfold higher than in the general population [49]. Both are chronic inflammatory diseases of the intestinal tract with similarities in their pathomechanisms and an overlap in their symptoms [54–56]. However, the pertinence of CD screening of IBD patients is still under debate and should be investigated [48, 50, 55, 57].

Similar to potential NCGS cases, irritable bowel syndrome (IBS) is clinically diagnosed and can be treated by food restriction [58, 59]. The clinical symptoms of IBS overlap with those associated with CD [60], and CD is 4-fold more common in IBS patients than in the healthy population and for this reason CD screening is recommended [61, 62]. Biesiekierski et al. showed for the first time in an Australian double-blinded randomized placebo-controlled trial that nonceliac IBS and therefore apparent NCGS patients benefited from a gluten-free diet [63]. However, further double-blinded crossover studies including a well-controlled low FODMAP diet showed no gluten-specific induced gastrointestinal symptoms in a comparable population [1]. In line with this, further similar studies by the same group corroborated these results and showed an association between gluten and mild depression assessed by the Spielberger State Trait Personality Inventory in non-CD IBS patients [2]. These findings support that gluten could trigger extraintestinal symptoms in some patients independent of the presence of enteropathy [64], but they do not necessarily support the coexistence of the so-called disorder NCGS with IBS. Consequently, further research is required to establish if coexistence of these two conditions truly exists or if NCGS is a subcondition of IBS.

## 4. Laboratory Tests for CD Diagnosis

### 4.1. Anti-Gliadin Antibodies (AGAs)

Although AGAs are generally present in the blood of CD patients [65, 66], they have also been reported in apparently healthy individuals, autoimmune or gastrointestinal diseases, schizophrenia, and “NCGS” [5, 6, 50, 66–69]. Therefore, AGAs alone do not discriminate between CD individuals and controls.

Although it has been reported that IgA-AGAs perform better than IgA anti-tissue transglutaminase 2 antibodies (IgA-tTG) and IgA anti-endomysium antibodies (IgA-EMA) in children younger than 18 months of age [70], a recent study showed only 5 out of 33 patients under 2 years of age with positive IgA-AGAs levels were confirmed histologically to have CD [71]. To avoid false negative results, some authors recommend endoscopy with small bowel biopsies to perform CD diagnosis in IgA-AGA positive individuals [72]. In fact, it is advisable to take intestinal biopsies in young children with severe symptoms of CD, even if serology is negative [21].

### 4.2. Anti-Endomysium Antibodies (EMA)

The first evidence that IgA-EMA could be used in CD diagnosis was given almost 30 years ago [73, 74]. Since then, several studies have evaluated the sensitivity and specificity of this immunofluorescence test using monkey esophagus or human umbilical cord as the tissue substrate. Generally, the cutoff values for a positive test equates to a serum dilution equal to or greater than 1 : 5 [75, 76] or 1 : 10 [77, 78].

The diagnostic accuracy of serological testing for CD has been previously reviewed [79, 80]. IgA-EMA sensitivity ranges were 90% to 98% in adult CD, with the highest value reached using monkey esophagus as the tissue substrate and for children, the sensitivity ranges were 93% to 97%, independent of tissue substrate [79, 80]. Notably, specificities were close to 100% in all cases, but specificities <95% in children younger than 2 years old have been reported when using monkey esophagus [81]. Overall, these studies show that false negative IgA-EMA results can occur and this has been attributed to a reduction of gluten intake or the use of immunosuppressant.

Although immunofluorescence tests are labor intensive and subject to interobserver variability, current ESPGHAN guidelines consider IgA-EMA antibodies as the standard reference for CD-specific antibody detection [21]. In fact, semiquantitative tests (rapid tests) should be corroborated by EMA or tTG ELISAs and those diagnostic tests for use in children should be validated with sera from at least 50 children with active CD and 100 control children of different ages against the reference of IgA-EMA positivity detected in an expert laboratory [21]. This task can be challenging in countries where CD is not commonly diagnosed. In such cases, the implementation of good laboratory practices can effectively help to reduce the rate of false negative/positive results.

### 4.3. Anti-Tissue Transglutaminase 2 Antibodies

In 1997 Dieterich et al. [82] found that tissue transglutaminase (tTG) is the main endomysial antigen (EMA). Based on this, a variety of ELISA tests to detect tTG-specific antibodies have been developed, the target antigens used include guinea pig or human tTG (recombinant or purified human tTG), and, notably, the choice of antigen can affect the performance of the tTG ELISA test. Moreover, there is evidence suggesting that IgA-tTG ELISAs perform better than their counterpart IgG-tTG in clinical setting [77, 83]. The mechanisms underlying the preferential production of IgA-tTG remain elusive, although it has been proposed that a group of tTG/gliadin-specific B cells are committed to be IgA-positive in well-established CD patients [84]. The production of IgA/IgG-tTG by B cells in the absence of tTG-specific T-cell “help” has been explained by Sollid et al. [85] as employing a hapten-carrier model. This model assumes the formation of tTG-gliadin immunocomplexes and subsequent recognition of these complexes by tTG-specific B cells. Further presentation of T-cell epitopes to gliadin-specific T-cells triggers the production of IgA/IgG-tTG.

**Figure 1:**
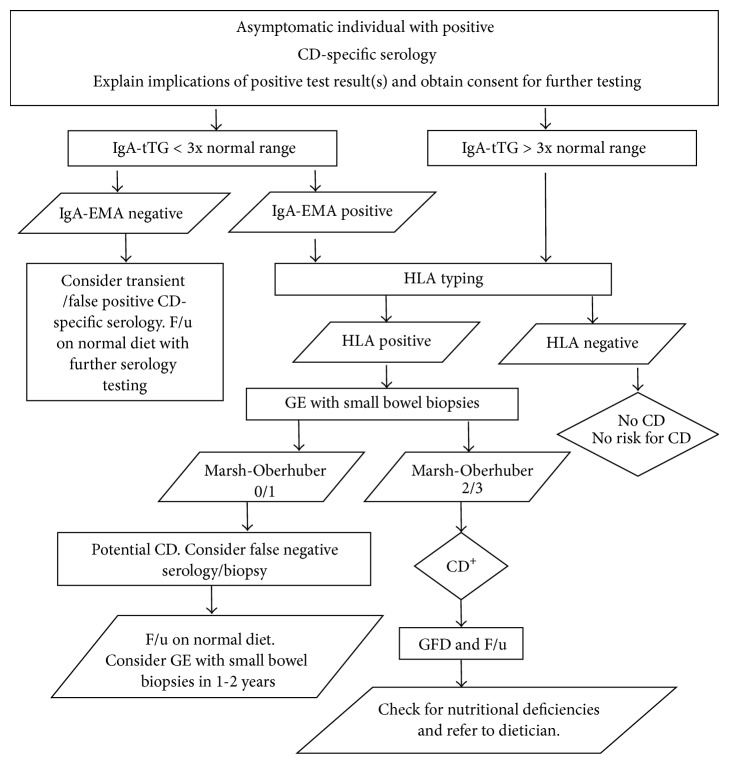
Proposed algorithm for CD diagnosis in asymptomatic individuals with positive CD-specific serology. GE: gastrointestinal endoscopy, GFD: gluten-free diet, and F/u: follow-up.

The reported sensitivities and specificities of IgA-tTG ELISAs employing guinea pig tTG as antigen were between 90% and 93% and 92.4% and 95%, respectively. Similarly, ELISAs employing human tTG have shown sensitivities and specificities between 94% and 98% and 95% and 99%, respectively [79, 80, 86]. Thus, IgA-tTG ELISAs using human tTG as antigen can be classified as the assays of choice for which a positive result should lead to endoscopy and small bowel biopsies to confirm CD [21, 87]. However, due to possible false positive results, it may be more convenient to retest by IgA-EMA serology in samples with values <3x normal range, especially in subclinical CD cases (Figure 1), rather than continuation onto invasive tests.

Children with subclinical CD but with positive IgA-tTG require special attention, as positivity (commonly <10x normal range) can be lost over time despite continuing gluten exposure [88]. This transient IgA-tTG has also been reported in children with type 1 diabetes mellitus [89]. Thus, in the absence of severe symptoms, serological follow-up is recommended before performing endoscopy with small bowel biopsies to confirm CD [88].

Hill and Holmes [90] and Dahlbom et al. [91] showed that in patients with signs and symptoms suggestive of CD and IgA-tTG levels >10x the normal range had a high likelihood for the presence of Marsh 3b or c villous atrophy. Though IgA-tTG performs better than IgG-tTG in children and adults [91], the IgG-tTG test remains relevant in IgA-deficient cases [21], which are relatively common in CD [92]. Although exceptions do remain [93], CD diagnosis can be performed without the need of intestinal biopsy in symptomatic children and adolescents with a tTG serology result >10x normal range [21]. This should be confirmed by EMA staining and HLA typing in a second blood sample to reinforce the diagnosis of CD [21]. It is important to note that different tTG ELISA kits used between different diagnostic laboratories can have varying results and/or interpretation of the results even analyzing the same sample [21].

### 4.4. Anti-Deamidated Gliadin Peptide (DGP) Antibodies

Some of the earliest evidence that deamidated gliadin peptides contain CD-relevant B cell epitopes was provided by Aleanzi et al. [94] and Schwertz et al. [95]. Currently, there are a large number of commercial anti-deamidated gliadin peptide ELISA tests available. This includes tests that detect IgA/IgG-DGPs individually or in combination with tTG. In general, DGP ELISAs have shown acceptable sensitivity and specificity compared to tTG ELISAs and EMA in both children and adults [83, 96–99].

In contrast to tTG ELISAs DGP assays seem to perform at similar levels independent of the isotype detected. In fact, there is a substantial difference between the generation of antibody isotypes against DGP and tTG [84]. The use of IgG-DGP ELISAs is advantageous in IgA-deficient individuals, which is a higher proportion in CD than the general population [92]. Supporting this, a diagnostic meta-analysis study has shown that IgA-tTG ELISA has greater diagnostic accuracy than IgA-DGPs (sensitivities of 93% versus 87% and specificities of 96% versus 94%, resp.) [80].

Numerous groups have evaluated the diagnostic accuracy of IgG-DGPs ELISAs. The results differ depending on the age of the population studied and the clinical setting. In general, sensitivities and specificities range between 65% and 95% and 81% and 100%, respectively [81, 83, 96, 97, 99]. Villalta et al. [100] reported that using IgG-DGPs ELISA detected up to 80% of the CD cases with selective IgA deficiency with a specificity of 98%. The same study reported sensitivities of 75% to 95% and specificities of 88% to 100% using different IgG-tTG ELISA kits.

We and others agree with the concept that the addition of an IgG ELISA assay could improve the accuracy for CD diagnosis [99]. Supporting this, it has been reported that in children <2 years old IgG-DGP ELISAs perform better than EMA tests and tTG ELISA. In this study sensitivities and specificities were 100% using 2 different IgG-DGP ELISA kits [81]. However, since it has been reported that some anti-DGP antibody positive children that are <2 years old became DGP antibody negative over time without maintaining a gluten-free diet, serological follow-up is recommended in this group of patients [101]. In general, due to the fact that performance of all CD-specific serology tests depends on the prevalence of the condition, the age of the subjects evaluated, and the amount of gluten ingested, these factors should be considered when interpreting CD-specific serology results.

### 4.5. Pathology Results (Biopsy Results)

Histological findings of CD are traditionally categorized according to three classifications: Marsh, Marsh/Oberhuber, and Corazza [23–27]. A detailed comparison of these histological classifications was provided by a panel of CD experts [22], recommending the Marsh/Oberhuber classification for reporting CD pathology results [21]. Previously, a count of ≥40 intraepithelial lymphocytes/100 enterocytes was considered to denote infiltrative changes, but this threshold was reduced to ≥25 intraepithelial lymphocytes/100 enterocytes due to its correlation with positive CD-specific serology and the possibility that higher thresholds could miss 50% of the cases [102].

According to current ESPGHAN guidelines, Marsh type 2 (normal architecture and infiltrative changes with crypt hyperplasia) or more severe intestinal lesions are considered CD-like enteropathy [21]. As other conditions share histopathological features of CD, such as allergies to proteins other than gluten, giardiasis, and collagenous sprue [103], consideration must be made to the clinical setting when interpreting pathology results. Furthermore, it should be considered that approximately 10% of patients presenting CD-like symptoms, positive CD-specific serology, and only infiltrative changes (potential CD) can benefit from a gluten-free diet [104].

Pathology reports should always include the following parameters: (1) description of sample orientation, (2) description of the villous (mild, moderate, or total atrophy) and crypt architecture, (3) villous/crypt ratio, and (4) number of intraepithelial lymphocytes [21]. It is recommended to include the Marsh/Oberhuber grade and suggest differential diagnosis or rebiopsy if necessary [103]. To be representative, biopsies must be taken when patients are on a gluten containing diet, and due to patchiness of the CD lesion [105, 106], at least four biopsies should be taken from the second/third portion of the duodenum, and at least one biopsy should be taken from the duodenal bulb for CD diagnosis [21, 87, 107–110].

### 4.6. HLA Typing

Genetic associations with CD include more than 39 non-HLA risk genes, but HLA genes provide the strongest genetic risk for CD [111]. The majority of CD patients express the HLA-DQ2.5 heterodimer encoded by HLA-DQB1^*^02 and DQA1^*^05 alleles. This is expressed either in* cis* on the DR3-DQ2.5 haplotype (DQB1^*^02:01, DQA1^*^05:01, and DRB1^*^03:01) or in* trans* (heterozygous for haplotypes DR5-DQ7 and DR7-DQ2.2), where the HLA-DQ2.5 heterodimer is encoded by DQB1^*^02:02 and DQA1^*^05:05. Low to intermediate risk for CD has been associated with both DR7-DQ2.2 (DQB1^*^02:02, DQA1^*^02:01, and DRB1^*^07) and heterozygous DR4-DQ8 (DQB1^*^03:02, DQA1^*^03, and DRB1^*^04). One of the first reports on the association between these haplotypes and CD was provided by Sollid et al. [112, 113]. Since then, several studies have supported this data and highlighted the involvement of other HLA haplotypes in CD susceptibility (Table 2).

Some studies have shown that more than 99% of CD patients carry genes that encode the HLA-DQ2.5, HLA-DQ2.2, and/or HLA-DQ8 heterodimers [28–30, 112]. In rare cases the disease predisposing HLA heterodimers are a result of a different combination of HLA alleles encoding dimers other than DQ2.5, DQ2.2, and DQ8. This includes expression of the DQA1^*^05:01 and DQB1^*^03:02 alleles that encode the HLA-DQ8.5 heterodimer in* trans*, present in approximately 1.6% of CD individuals [28]. In addition, the HLA-DQ2.3 heterodimer is encoded in* trans* by the DQA1^*^03:01 and DQB1^*^02:01 and the HLA-DQ9 heterodimer is encoded by the DQA1^*^03 and DQB1^*^03:03 alleles. Notably, it has been shown that these HLA-DQ heterodimers on antigen presenting cells can load and present gluten peptides to T-cells found in CD patients [31–33]; however data on the frequency of these haplotypes in CD are limited.

**Figure 2:**
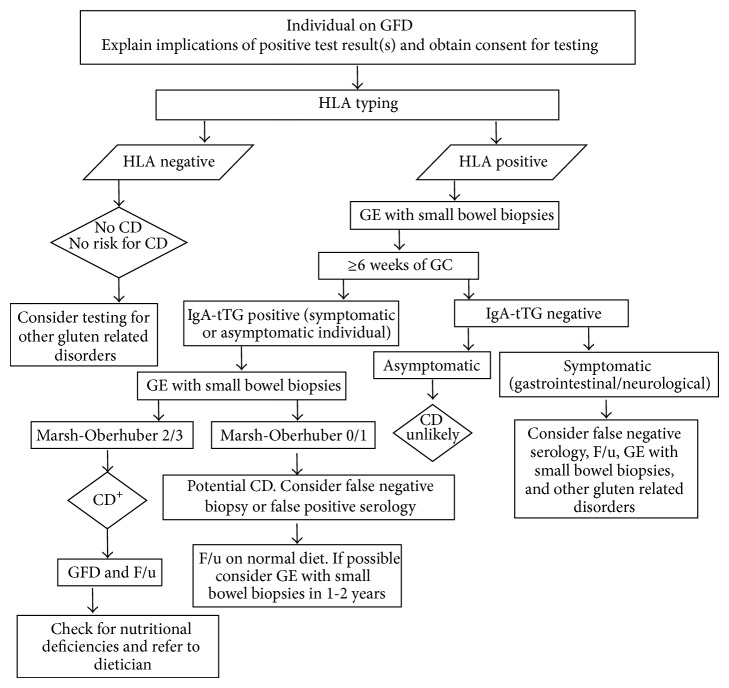
Proposed algorithm for CD diagnosis in patients already following gluten-free diet. GE: gastrointestinal endoscopy, GC: gluten challenge, GFD: gluten-free diet, and F/u: follow-up.

Due to its high negative predictive value, the main utility of HLA typing is to rule out CD. This is of particular relevance when assessing CD or other gluten related disorders in patients already following gluten-free diet (Figure 2). Current ESPGHAN guidelines suggest that HLA typing should be done by DNA testing for the four alleles encoding the DQ2.5 and DQ8 heterodimers (DQA1^*^05, DQB1^*^02, DQA1^*^03, and DQB1^*^03:02) [21]. Furthermore, we suggest to additionally test for the DQA1^*^02:01 allele as the DR7-DQ2.2 haplotype can be found in around 4% of DQA1^*^05, DQB1^*^02, DQA1^*^03, and DQB1^*^03:02 negative CD individuals [28].

Some laboratories that perform CD-associated HLA-DQ genetic testing only report the presence of the DQB1^*^02 and DQB1^*^03:02 alleles. This is reasonable as DQB1^*^02 is the major allele associated with CD and DQB1^*^03:02 is always found with DQA1^*^03 [34]. Although this strategy reduces costs of the HLA typing, it has been reported that a small proportion of CD patients were carrying just the DQA1∗05 genetic risk allele [28, 30], and as previously mentioned, some patients carry just the DR7-DQ2.2 haplotype. Therefore, ideally the full DR3-DQ2.5, DR7-DQ2.2, and DR4-DQ8 genotype should be performed and reported including whether the patients are homozygous/heterozygous. In addition, the inclusion of the relative genetic risk for CD would aid interpretation of the results (Table 2).

## 5. Patients Already Following Gluten-Free Diet

Although discouraged in children under the age of 5 years and during their pubertal growth spurt [21], gluten challenge is recommended in individuals following a gluten-free diet without proper diagnostic work-up of CD, in order to confirm the condition (Figure 2). The biggest limitation to gluten challenge protocols is that symptomatic relapse often precedes serological and histological relapse. To overcome this, some studies have evaluated the CD-specific serology and histology response to gluten challenge employing different amounts of gluten and timeframe [76, 114–119]. These studies have employed between 2.5 and 7.5 g of gluten daily for at least 2 weeks but the histological changes are highly variable, limiting the use of this approach. With regard to serological response, it has been shown that less than 50% of CD cases on remission seroconverted from negative to positive when eating 1 to 5 g of gluten daily for more than 4 weeks [76].

Current ESPGHAN guidelines recommend a gluten intake of at least 15 g of gluten daily to perform gluten challenge [21] and the American Gastroenterological Association 2006 technical review recommends this practice for at least 4 weeks [88]. Certainly, some clinicians commonly perform gluten challenge for 6 weeks or longer. The patient will be considered to have relapsed disease if CD-associated serology becomes positive and a clinical and/or histological relapse is observed [21] (Figure 2).

New diagnostic approaches that avoid prolonged gluten challenges in patients already following gluten-free diet are needed. Finding by Anderson et al. [120] describing the ability to detect gluten-specific T-cells in peripheral blood of treated HLA-DQ2.5 CD individuals six days after they had started a 3-day gluten challenge has led to the potential of T-cell based diagnostics. Further characterization of the immunodominant gluten T-cell epitopes recognized by peripheral blood T-cells was valuable and allows the design and testing of new diagnostic and therapeutic approaches [121–125]. Moreover, Ontiveros et al. [126] have recently designed and tested a peptide-based whole-blood ELISA diagnostic test based on the 3-day gluten challenge. The test could potentially discriminate between HLA-DQ2.5 CD and HLA-DQ2.5 individuals on gluten-free diet that fit most of the proposed NCGS definitions. Similarly, an* ex vivo* gliadin challenge of small bowel biopsies has been proposed to identify difficult to diagnose CD patients [127]. These tests are in their infancy and they require validation with larger cohorts including HLA-matched controls.

## 6. Conclusions

Although some CD research groups have stated their position on the terms used to categorize CD subtypes, there is still a gap to be filled and a need for consensus in this field. Until the scientific community accepts the use of one terminology, it will be important for authors to clearly state their definition of terms employed to describe CD subtypes. This also applies to the use of the acronym “NCGS,” which seems to have been accepted by the scientific community, based on published papers. Motivated by recent definitions of CD and other gluten related disorders, we are aligned to the adoption of the terms classical, nonclassical, subclinical, and potential CD to define CD subtypes. Although intestinal and extraintestinal symptoms commonly overlap, the presence of gastrointestinal symptoms means a classical CD subtype.

CD is a condition relatively difficult to diagnose that shares clinical and histological characteristics with other gastrointestinal diseases. CD-specific serology tests are useful diagnostic tools to discriminate between CD and other gastrointestinal conditions. Therefore, due to the variety of diagnostic kits available, both general practitioners and medical specialists should be aware of the diagnostic performances of these kits in different clinical settings. The combination of IgA-tTG and IgG-DGP measurements seems to be appropriate in patients on a gluten containing diet. HLA typing in conjunction with CD-specific serology has become popular in the diagnostic work-up of CD, and with such an approach, it is possible to diagnose CD without performing gastrointestinal endoscopy with small bowel biopsies in some children. In young children with isolated IgA-AGA or severe symptoms of CD it is advisable to take intestinal biopsies to avoid false negative/positive results. In the case of HLA positive patients already following gluten-free diet, a prolonged gluten challenge is still required. However, symptomatic relapse often precedes histological and/or serological relapse, making prolonged gluten challenge unacceptable for the majority of the patients. This is an area that requires further research to develop a less invasive and well tolerated diagnostic test.

The literature suggests that FODMAPs and not gluten* per se* are the triggers of gastrointestinal symptoms in patients that fit most of the proposed NCGS definitions. Interestingly, wheat, rye, and barley are food sources of FODMAPs and should be avoided in FODMAP sensitive individuals. Finally, there is a strong clinical need for biomarkers in the diagnostic work-up of “NCGS.” The availability of sensitive and specific biomarkers will help clarify whether this disorder coexists with other gastrointestinal conditions. Meanwhile, diagnosis of “NCGS” should only occur after CD, wheat allergies, and other inflammatory disorders have been ruled out, including sensitivity to nongluten food constituents from wheat that can trigger gastrointestinal symptoms.

## Figures and Tables

**Table 1:** Classification of CD subtypes.

CD subtype	Symptoms	Serology^+^/HLA^++^	Pathology classification		
			Marsh	Marsh-Oberhuber	Corazza
Classical	Gastrointestinal symptoms and signs (diarrhea, abdominal distension, constipation, and abdominal pain)	+/+	Type 2/3^*^	Type 2/3a, b or c^*^	Grade A/B1 or B2^*^

Nonclassical	Extraintestinal symptoms and signs (anemia, neuropathy, osteoporosis, and short stature)	+/+	Type 2/3	Type 2/3a, b or c	Grade A/B1 or B2

Subclinical	Asymptomatic	+/+	Type 2/3	Type 2/3a, b or c	Grade A/B1 or B2

Potential	Presence or absence of symptoms	+/+	Type 0/1^**^	Type 0/1^**^	Normal/Grade A^**^

^+^positive CD-specific serology (mainly IgA-EMA, IgA-tTG, and/or IgG-DGP).

^++^Presence of genes/haplotype associated with CD (see Table 2).

^*^Intraepithelial lymphocytosis (≥25/100 enterocytes) with crypt hyperplasia (Marsh-Oberhuber type 2) and partial/total villous atrophy.

^**^Normal architecture/intraepithelial lymphocytosis.

References [21–27].

**Table 2:** HLA genetics and risk associated with CD.

HLA-DQA1 alleles	HLA-DQB1 alleles	HLA-DQ heterodimers	Predisposition for CD^+^
^*^05:01, ^*^05:01	^*^02:01, ^*^02:01	DQ2.5 (homozygous)	Very high
^*^05:01, ^*^03	^*^02:01, ^*^03:02	DQ2.5/DQ8/DQ2.3/DQ8.5	Very high
^*^05:01, ^*^02:01	^*^02:01, ^*^02:02	DQ2.5/DQ2.2 (encoded in cis and trans)	Very high
^*^05:01, x	^*^02:01, ^*^02	DQ2.5 (encoded in cis and trans)	Very high
^*^05:01, x	^*^02:01, x	DQ2.5 (heterozygous)	High
^*^05:05, ^*^02:01	^*^03:01, ^*^02:02	DQ2.5 (encoded in trans)/DQ2.2	High
^*^03, ^*^03	^*^03:02, ^*^03:02	DQ8 (homozygous)	High
^*^03, ^*^02:01	^*^03:02, ^*^02:02	DQ8/DQ2.2/DQ2.3	High
^*^03, x	^*^03:02, ^*^02	DQ8/DQ2.3	High
^*^03, x	^*^03:02, x	DQ8 (heterozygous)	Intermediate
^*^02:01, ^*^02:01	^*^02:02, ^*^02:02	DQ2.2 (homozygous)	Intermediate
^*^02:01, x	^*^02:02, ^*^02	DQ2.2 (encoded in cis and trans)	Intermediate
x, x	^*^02:01, ^*^02:01	Half DQ2.5	Intermediate
^*^02:01, x	^*^02:02, x	DQ2.2 (heterozygous)	Low
x, x	^*^02:01	Half DQ2.5	Low
^*^05:01	x, x	Half DQ2.5	Low
^*^03:01, x	^*^02:01, x	DQ2.3/x	ND^++, #^
^*^05, x	^*^03:02, x	DQ8.5/x	ND
^*^03, x	^*^03:03, x	DQ9/x	ND

^+^Predisposition for CD is based on the prevalence of genes/haplotypes and the immune recognition of CD-associated gluten T-cell epitopes [28–38].

^++^ND; nondetermined.

^#^It is possible that some HLA-DQ2.5 “restricted” gluten T-cell epitopes can be loaded and presented by the heterodimer DQ2.3 [31].

x denotes a non-CD related genotype.

## References

[1] Biesiekierski J. R., Peters S. L., Newnham E. D., Rosella O., Muir J. G., Gibson P. R. (2013). No effects of gluten in patients with self-reported non-celiac gluten sensitivity after dietary reduction of fermentable, poorly absorbed, short-chain carbohydrates. *Gastroenterology*.

[2] Peters S. L., Biesiekierski J. R., Yelland G. W., Muir J. G., Gibson P. R. (2014). Randomised clinical trial: gluten may cause depression in subjects with non-coeliac gluten sensitivity—an exploratory clinical study. *Alimentary Pharmacology and Therapeutics*.

[3] Junker Y., Zeissig S., Kim S.-J. (2012). Wheat amylase trypsin inhibitors drive intestinal inflammation via activation of toll-like receptor 4. *Journal of Experimental Medicine*.

[4] Genuis S. J., Lobo R. A. (2014). Gluten sensitivity presenting as a neuropsychiatric disorder. *Gastroenterology Research and Practice*.

[5] Volta U., Tovoli F., Cicola R. (2012). Serological tests in gluten sensitivity (nonceliac gluten intolerance). *Journal of Clinical Gastroenterology*.

[6] Volta U., de Giorgio R. (2012). New understanding of gluten sensitivity. *Nature Reviews Gastroenterology and Hepatology*.

[7] Kaukinen K., Lindfors K., Collin P., Koskinen O., Mäki M. (2010). Coeliac disease—a diagnostic and therapeutic challenge. *Clinical Chemistry and Laboratory Medicine*.

[8] Stazi A. V., Trecca A., Trinti B. (2008). Osteoporosis in celiac disease and in endocrine and reproductive disorders. *World Journal of Gastroenterology*.

[9] Emami M. H., Karimi S., Kouhestani S. (2012). Is routine duodenal biopsy necessary for the detection of celiac disease in patients presenting with iron deficiency anemia?. *International Journal of Preventive Medicine*.

[10] Bansal D., Trehan A., Gupta M. K., Varma N., Marwaha R. K. (2011). Serodiagnosis of celiac disease in children referred for evaluation of anemia: a pediatric hematology unit's experience. *Indian Journal of Pathology and Microbiology*.

[11] Catassi C., Fasano A. (2004). Celiac disease as a cause or growth retardation in childhood. *Current Opinion in Pediatrics*.

[12] Pietzak M. (2012). Celiac disease, wheat allergy, and gluten sensitivity: when gluten free is not a fad. *Journal of Parenteral and Enteral Nutrition*.

[13] Sapone A., Bai J. C., Ciacci C. (2012). Spectrum of gluten-related disorders: consensus on new nomenclature and classification. *BMC Medicine*.

[14] Kabbani T. A., Vanga R. R., Leffler D. A. (2014). Celiac disease or non-celiac gluten sensitivity? An approach to clinical differential diagnosis. *American Journal of Gastroenterology*.

[15] Emami M. H., Kouhestani S., Karimi S. (2012). Frequency of celiac disease in adult patients with typical or atypical malabsorption symptoms in Isfahan, Iran. *Gastroenterology Research and Practice*.

[16] Kochhar R., Jain K., Thapa B. R. (2012). Clinical presentation of celiac disease among pediatric compared to adolescent and adult patients. *Indian Journal of Gastroenterology*.

[17] Roma E., Panayiotou J., Karantana H. (2009). Changing pattern in the clinical presentation of pediatric celiac disease: a 30-year study. *Digestion*.

[18] Balamtekin N., Uslu N., Baysoy G. (2010). The presentation of celiac disease in 220 Turkish children. *Turkish Journal of Pediatrics*.

[19] Admou B., Essaadouni L., Krati K. (2012). Atypical celiac disease: from recognizing to managing. *Gastroenterology Research and Practice*.

[20] Ganji A., Esmaielzadeh A., Aghayee M. A., Goshayeshi L., Ghaffarzadegan K. (2014). The clinical presentation of celiac disease: experiences from Northeastern Iran. *Middle East Journal of Digestive Diseases*.

[21] Husby S., Koletzko S., Korponay-Szabó I. R. (2012). European society for pediatric gastroenterology, hepatology, and nutrition guidelines for the diagnosis of coeliac disease. *Journal of Pediatric Gastroenterology and Nutrition*.

[22] Ludvigsson J. F., Leffler D. A., Bai J. C. (2013). The Oslo definitions for coeliac disease and related terms. *Gut*.

[23] Marsh M. N., Bjarnason I., Shaw J., Ellis A., Baker R., Peters T. J. (1990). Studies of intestinal lymphoid tissue. XIV-HLA status, mucosal morphology, permeability and epithelial lymphocyte populations in first degree relatives of patients with coeliac disease. *Gut*.

[24] Marsh M. N. (1992). Gluten, major histocompatibility complex, and the small intestine: a molecular and immunobiologic approach to the spectrum of gluten sensitivity (‘celiac sprue’). *Gastroenterology*.

[25] Oberhuber G., Granditsch G., Vogelsang H. (1999). The histopathology of coeliac disease: Time for a standardized report scheme for pathologists. *European Journal of Gastroenterology and Hepatology*.

[26] Corazza G. R., Villanacci V. (2005). Coeliac disease. *Journal of Clinical Pathology*.

[27] Corazza G. R., Villanacci V., Zambelli C. (2007). Comparison of the interobserver reproducibility with different histologic criteria used in celiac disease. *Clinical Gastroenterology and Hepatology*.

[28] Karell K., Louka A. S., Moodie S. J. (2003). HLA types in celiac disease patients not carrying the *DQA1^∗^05-DQB1^∗^02* (DQ2) heterodimer: results from the European Genetics Cluster on Celiac Disease. *Human Immunology*.

[29] Alarida K., Harown J., Di Pierro M. R., Drago S., Catassi C. (2010). HLA-DQ2 and -DQ8 genotypes in celiac and healthy Libyan children. *Digestive and Liver Disease*.

[30] Megiorni F., Mora B., Bonamico M. (2009). HLA-DQ and risk gradient for celiac disease. *Human Immunology*.

[31] Tollefsen S., Hotta K., Chen X. (2012). Structural and functional studies of *trans*-encoded HLA-DQ2.3 (DQA1^∗^03:01/DQB1^∗^02:01) protein molecule. *The Journal of Biological Chemistry*.

[32] Kooy-Winkelaar Y., Van Lummel M., Moustakas A. K. (2011). Gluten-specific T cells cross-react between HLA-DQ8 and the HLA-DQ2*α*/DQ8*β* transdimer. *Journal of Immunology*.

[33] Bodd M., Tollefsen S., Bergseng E., Lundin K. E. A., Sollid L. M. (2012). Evidence that HLA-DQ9 confers risk to celiac disease by presence of DQ9-restricted gluten-specific T cells. *Human Immunology*.

[34] Margaritte-Jeannin P., Babron M. C., Bourgey M. (2004). HLA-DQ relative risks for coeliac disease in European populations: a study of the European Genetics Cluster on Coeliac Disease. *Tissue Antigens*.

[35] Bodd M., Kim C., Lundin K. E. A., Sollid L. M. (2012). T-cell response to gluten in patients with HLA-DQ2.2 reveals requirement of peptide-MHC stability in celiac disease. *Gastroenterology*.

[36] Fallang L.-E., Bergseng E., Hotta K., Berg-Larsen A., Kim C.-Y., Sollid L. M. (2009). Differences in the risk of celiac disease associated with HLA-DQ2.5 or HLA-DQ2.2 are related to sustained gluten antigen presentation. *Nature Immunology*.

[37] Ploski R., Ek J., Thorsby E., Sollid L. M. (1993). On the HLA-DQ(*α*1^*^0501, *β*1^*^0201)-associated susceptibility in celiac disease: a possible gene dosage effect of DQB1^*^0201. *Tissue Antigens*.

[38] Qiao S.-W., Iversen R., Ráki M., Sollid L. M. (2012). The adaptive immune response in celiac disease. *Seminars in Immunopathology*.

[39] Ferguson A., Arranz E., O'Mahony S. (1993). Clinical and pathological spectrum of coeliac disease—active, silent, latent, potential. *Gut*.

[40] Troncone R., Jabri B. (2011). Coeliac disease and gluten sensitivity. *Journal of Internal Medicine*.

[41] Sapone A., Lammers K. M., Casolaro V. (2011). Divergence of gut permeability and mucosal immune gene expression in two gluten-associated conditions: celiac disease and gluten sensitivity. *BMC Medicine*.

[42] Mäkelä M. J., Eriksson C., Kotaniemi-Syrjänen A. (2014). Wheat allergy in children—new tools for diagnostics. *Clinical & Experimental Allergy*.

[43] Biesiekierski J. R., Muir J. G., Gibson P. R. (2013). Is gluten a cause of gastrointestinal symptoms in people without celiac disease?. *Current Allergy and Asthma Reports*.

[44] Ontiveros N., Flores-Mendoza L. K., Canizalez-Román V. A., Cabrera-Chávez F. (2014). Food allergy: prevalence and food technology approaches for the control of IgE-mediated food allergy. *Austin Journal of Nutrition and Food Sciences*.

[45] Bizzaro N., Tozzoli R., Villalta D., Fabris M., Tonutti E. (2012). Cutting-Edge issues in celiac disease and in gluten intolerance. *Clinical Reviews in Allergy and Immunology*.

[46] Kotaniemi-Syrjänen A., Palosuo K., Jartti T., Kuitunen M., Pelkonen A. S., Mäkelä M. J. (2010). The prognosis of wheat hypersensitivity in children. *Pediatric Allergy and Immunology*.

[47] Vojdani A., Perlmutter D. (2013). Differentiation between celiac disease, nonceliac gluten sensitivity, and their overlapping with Crohn's disease: a case series. *Case Reports in Immunology*.

[48] Mantzaris G. J., Roussos A., Koilakou S. (2005). Prevalence of celiac disease in patients with Crohn's disease. *Inflammatory Bowel Diseases*.

[49] Leeds J. S., Höroldt B. S., Sidhu R. (2007). Is there an association between coeliac disease and inflammatory bowel diseases? A study of relative prevalence in comparison with population controls. *Scandinavian Journal of Gastroenterology*.

[50] Tursi A., Giorgetti G. M., Brandimarte G., Elisei W. (2005). High prevalence of celiac disease among patients affected by Crohn's disease. *Inflammatory Bowel Diseases*.

[51] Yang A., Chen Y., Scherl E., Neugut A. I., Bhagat G., Green P. H. R. (2005). Inflammatory bowel disease in patients with celiac disease. *Inflammatory Bowel Diseases*.

[52] Patel J., Agasti A., Rao S., Srinivas M. G., Patel M., Sawant P. (2011). Celiac disease preceding Crohn's disease?. *Tropical Gastroenterology*.

[53] Oxford E. C., Nguyen D. D., Sauk J. (2013). Impact of coexistent celiac disease on phenotype and natural history of inflammatory bowel diseases. *American Journal of Gastroenterology*.

[54] Festen E. A. M., Szperl A. M., Weersma R. K., Wijmenga C., Wapenaar M. C. (2009). Inflammatory bowel disease and celiac disease: overlaps in the pathology and genetics, and their potential drug targets. *Endocrine, Metabolic and Immune Disorders—Drug Targets*.

[55] Pascual V., Dieli-Crimi R., López-Palacios N., Bodas A., Medrano L. M., Núñez C. (2014). Inflammatory bowel disease and celiac disease: overlaps and differences. *World Journal of Gastroenterology*.

[56] Häuser W., Janke K.-H., Klump B., Gregor M., Hinz A. (2010). Anxiety and depression in adult patients with celiac disease on a gluten-free diet. *World Journal of Gastroenterology*.

[57] Tursi A., Giorgetti G. M., Brandimarte G., Elisei W. (2006). Crohn's disease and celiac disease: association or epiphenomenon?. *European Review for Medical and Pharmacological Sciences*.

[58] Wahnschaffe U., Schulzke J.-D., Zeitz M., Ullrich R. (2007). Predictors of clinical response to gluten-free diet in patients diagnosed with diarrhea-predominant irritable bowel syndrome. *Clinical Gastroenterology and Hepatology*.

[59] Halmos E. P., Power V. A., Shepherd S. J., Gibson P. R., Muir J. G. (2014). A diet low in FODMAPs reduces symptoms of irritable bowel syndrome. *Gastroenterology*.

[60] Verdu E. F., Armstrong D., Murray J. A. (2009). Between celiac disease and irritable bowel syndrome: the ‘
no man's land’ of gluten sensitivity. *American Journal of Gastroenterology*.

[61] O'Leary C., Wieneke P., Buckley S. (2002). Celiac disease and irritable bowel-type symptoms. *American Journal of Gastroenterology*.

[62] Cristofori F., Fontana C., Magistà A. (2014). Increased prevalence of celiac disease among pediatric patients with irritable bowel syndrome: a 6-year prospective cohort study. *JAMA Pediatrics*.

[63] Biesiekierski J. R., Newnham E. D., Irving P. M. (2011). Gluten Causes gastrointestinal symptoms in subjects without celiac disease: a double-blind randomized placebo-controlled trial. *American Journal of Gastroenterology*.

[64] Hadjivassiliou M., Kandler R. H., Chattopadhyay A. K. (2006). Dietary treatment of gluten neuropathy. *Muscle and Nerve*.

[65] Troncone R., Ferguson A. (1991). Anti-gliadin antibodies. *Journal of Pediatric Gastroenterology and Nutrition*.

[66] ten Dam M., van de Wal Y., Mearin M. L. (1998). Anti-alpha-gliadin antibodies (AGA) in the serum of coeliac children and controls recognize an identical collection of linear epitopes of alpha-gliadin. *Clinical & Experimental Immunology*.

[67] Ribes Coninckx C., Giliams J. P., Polanco I., Pena A. S. (1984). IgA antigliadin antibodies in celiac and inflammatory bowel disease. *Journal of Pediatric Gastroenterology and Nutrition*.

[68] George E. K., Mearin M. L., Bouquet J. (1996). Screening for coeliac disease in Dutch children with associated diseases. *Acta Paediatrica*.

[69] Jackson J., Eathon W., Cascella N. (2014). Gluten sensitivity and relationship to psychiatric symptoms in people with schizophrenia. *Schizophrenia Research*.

[70] Lagerqvist C., Dahlbom I., Hansson T. (2008). Antigliadin immunoglobulin a best in finding celiac disease in children younger than 18 months of age. *Journal of Pediatric Gastroenterology and Nutrition*.

[71] Foucher B., Johanet C., Jégo-Desplat S. (2012). Are immunoglobulin a anti-gliadin antibodies helpful in diagnosing coeliac disease in children younger than 2 years?. *Journal of Pediatric Gastroenterology and Nutrition*.

[72] Villanacci V., Ceppa P., Tavani E., Vindigni C., Volta U. (2011). Coeliac disease: the histology report. *Digestive and Liver Disease*.

[73] Chorzelski T. P., Beutner E. H., Sulej J. (1984). IgA anti-endomysium antibody. A new immunological marker of dermatitis herpetiformis and coeliac disease. *British Journal of Dermatology*.

[74] Chorzelski T. P., Sulej J., Tchorzewska H., Jablonska S., Beutner E. H., Kumar V. (1983). IgA class endomysium antibodies in dermatitis herpetiformis and coeliac disease. *Annals of the New York Academy of Sciences*.

[75] Sulkanen S., Halttunen T., Laurila K. (1998). Tissue transglutaminase autoantibody enzyme-linked immunosorbent assay in detecting celiac disease. *Gastroenterology*.

[76] Lähdeaho M.-L., Mäki M., Laurila K., Huhtala H., Kaukinen K. (2011). Small-bowel mucosal changes and antibody responses after low- and moderate-dose gluten challenge in celiac disease. *BMC Gastroenterology*.

[77] Parizade M., Bujanover Y., Weiss B., Nachmias V., Shainberg B. (2009). Performance of serology assays for diagnosing celiac disease in a clinical setting. *Clinical and Vaccine Immunology*.

[78] Lionetti E., Castellaneta S., Pulvirenti A. (2012). Prevalence and natural history of potential celiac disease in at-family-risk infants prospectively investigated from birth. *Journal of Pediatrics*.

[79] Rostom A., Dubé C., Cranney A. (2005). The diagnostic accuracy of serologic tests for celiac disease: a systematic review. *Gastroenterology*.

[80] Lewis N. R., Scott B. B. (2010). Meta-analysis: deamidated gliadin peptide antibody and tissue transglutaminase antibody compared as screening tests for coeliac disease. *Alimentary Pharmacology & Therapeutics*.

[81] Mubarak A., Gmelig-Meyling F., Wolters V., Ten Kate F., Houwen R. (2011). Immunoglobulin G antibodies against deamidated-gliadin-peptides outperform anti-endomysium and tissue transglutaminase antibodies in children <2 years age. *APMIS*.

[82] Dieterich W., Ehnis T., Bauer M. (1997). Identification of tissue transglutaminase as the autoantigen of celiac disease. *Nature Medicine*.

[83] Rashtak S., Ettore M. W., Homburger H. A., Murray J. A. (2008). Comparative usefulness of deamidated gliadin antibodies in the diagnosis of celiac disease. *Clinical Gastroenterology and Hepatology*.

[84] Marietta E. V., Rashtak S., Murray J. A. (2009). Correlation analysis of celiac sprue tissue transglutaminase and deamidated gliadin IgG/IgA. *World Journal of Gastroenterology*.

[85] Sollid L. M., Molberg Ø., Mcadam S., Lundin K. E. A. (1997). Autoantibodies in coeliac disease: tissue transglutaminase guilt by association?. *Gut*.

[86] Zintzaras E., Germenis A. E. (2006). Performance of antibodies against tissue transglutaminase for the diagnosis of celiac disease: meta-analysis. *Clinical and Vaccine Immunology*.

[87] Rostom A., Murray J. A., Kagnoff M. F. (2006). American Gastroenterological Association (AGA) Institute technical review on the diagnosis and management of celiac disease. *Gastroenterology*.

[88] Simell S., Hoppu S., Hekkala A. (2007). Fate of five celiac disease-associated antibodies during normal diet in genetically at-risk children observed from birth in a natural history study. *The American Journal of Gastroenterology*.

[89] Waisbourd-Zinman O., Rosenbach Y., Shalitin S. (2012). Spontaneous normalization of anti-tissue transglutaminase antibody levels is common in children with type 1 diabetes mellitus. *Digestive Diseases and Sciences*.

[90] Hill P. G., Holmes G. K. T. (2008). Coeliac disease: a biopsy is not always necessary for diagnosis. *Alimentary Pharmacology and Therapeutics*.

[91] Dahlbom I., Korponay-Szabó I. R., Kovács J. B., Szalai Z., Mäki M., Hansson T. (2010). Prediction of clinical and mucosal severity of coeliac disease and dermatitis herpetiformis by quantification of IgA/IgG serum antibodies to tissue transglutaminase. *Journal of Pediatric Gastroenterology and Nutrition*.

[92] Cataldo F., Marino V., Ventura A., Bottaro G., Corazza G. R. (1998). Prevalence and clinical features of selective immunoglobulin A deficiency in coeliac disease: an Italian multicentre study. Italian Society of Paediatric Gastroenterology and Hepatology (SIGEP) and ‘Club del Tenue’ Working Groups on Coeliac Disease. *Gut*.

[93] Schirru E., Jores R.-D., Congia M. (2014). Prudence is necessary in the application of the new ESPGHAN criteria for celiac disease omitting duodenal biopsy: a case report. *European Journal of Gastroenterology and Hepatology*.

[94] Aleanzi M., Demonte A. M., Esper C., Garcilazo S., Waggener M. (2001). Antibody recognition against native and selectively deamidated gliadin peptides. *Clinical Chemistry*.

[95] Schwertz E., Kahlenberg F., Sack U. (2004). Serologic assay based on gliadin-related nonapeptides as a highly sensitive and specific diagnostic aid in celiac disease. *Clinical Chemistry*.

[96] Sugai E., Vázquez H., Nachman F. (2006). Accuracy of testing for antibodies to synthetic gliadin-related peptides in celiac disease. *Clinical Gastroenterology and Hepatology*.

[97] Agardh D. (2007). Antibodies against synthetic deamidated gliadin peptides and tissue transglutaminase for the identification of childhood celiac disease. *Clinical Gastroenterology and Hepatology*.

[98] Kaukinen K., Collin P., Laurila K., Kaartinen T., Partanen J., Mäki M. (2007). Resurrection of gliadin antibodies in coeliac disease. Deamidated gliadin peptide antibody test provides additional diagnostic benefit. *Scandinavian Journal of Gastroenterology*.

[99] Volta U., Granito A., Fiorini E. (2008). Usefulness of antibodies to deamidated gliadin peptides in celiac disease diagnosis and follow-up. *Digestive Diseases and Sciences*.

[100] Villalta D., Alessio M. G., Tampoia M. (2007). Testing for IgG class antibodies in celiac disease patients with selective IgA deficiency. A comparison of the diagnostic accuracy of 9 IgG anti-tissue transglutaminase, 1 IgG anti-gliadin and 1 IgG anti-deaminated gliadin peptide antibody assays. *Clinica Chimica Acta*.

[101] Parizade M., Shainberg B. (2010). Positive deamidated gliadin peptide antibodies and negative tissue transglutaminase IgA antibodies in a pediatric population: to biopsy or not to biopsy. *Clinical and Vaccine Immunology*.

[102] Walker M. M., Murray J. A., Ronkainen J. (2010). Detection of celiac disease and lymphocytic enteropathy by parallel serology and histopathology in a population-based study. *Gastroenterology*.

[103] Dickson B. C., Streutker C. J., Chetty R. (2006). Coeliac disease: an update for pathologists. *Journal of Clinical Pathology*.

[104] Kakar S., Pardi D. S., Burgart L. J. (2003). Colonic ulcers accompanying collagenous colitis: implication of nonsteroidal anti-inflammatory drugs. *American Journal of Gastroenterology*.

[105] Kurien M., Evans K. E., Hopper A. D., Hale M. F., Cross S. S., Sanders D. S. (2012). Duodenal bulb biopsies for diagnosing adult celiac disease: is there an optimal biopsy site?. *Gastrointestinal Endoscopy*.

[106] Ravelli A., Villanacci V., Monfredini C., Martinazzi S., Grassi V., Manenti S. (2010). How patchy is patchy villous atrophy? Distribution pattern of histological lesions in the duodenum of children with celiac disease. *American Journal of Gastroenterology*.

[107] Hopper A. D., Cross S. S., Sanders D. S. (2008). Patchy villous atrophy in adult patients with suspected gluten-sensitive enteropathy: is a multiple duodenal biopsy strategy appropriate?. *Endoscopy*.

[108] Gonzalez S., Gupta A., Cheng J. (2010). Prospective study of the role of duodenal bulb biopsies in the diagnosis of celiac disease. *Gastrointestinal Endoscopy*.

[109] Nenna R., Pontone S., Pontone P. (2012). Duodenal bulb in celiac adults: the ‘whether biopsying’ dilemma. *Journal of Clinical Gastroenterology*.

[110] Rashid M., MacDonald A. (2009). Importance of duodenal bulb biopsies in children for diagnosis of celiac disease in clinical practice. *BMC Gastroenterology*.

[111] Trynka G., Wijmenga C., van Heel D. A. (2010). A genetic perspective on coeliac disease. *Trends in Molecular Medicine*.

[112] Sollid L. M., Markussen G., Ek J., Gjerde H., Vartdal F., Thorsby E. (1989). Evidence for a primary association of celiac disease to a particular HLA-DQ *α*/*β* heterodimer. *Journal of Experimental Medicine*.

[113] Sollid L. M., Thorsby E. (1993). HLA susceptibility genes in celiac disease: genetic mapping and role in pathogenesis. *Gastroenterology*.

[114] Montgomery A. M. P., Goka A. K. J., Kumar P. J., Farthing M. J. G., Clark M. L. (1988). Low gluten diet in the treatment of adult coeliac disease: effect on jejunal morphology and serum anti-gluten antibodies. *Gut*.

[115] Kumar P. J., O'Donoghue D. P., Stenson K., Dawson A. M. (1979). Reintroduction of gluten in adults and children with treated coeliac disease. *Gut*.

[116] Catassi C., Rossini M., Ratsch I.-M. (1993). Dose dependent effects of protracted ingestion of small amounts of gliadin in coeliac disease children: A clinical and jejunal morphometric study. *Gut*.

[117] Catassi C., Fabiani E., Iacono G. (2007). A prospective, double-blind, placebo-controlled trial to establish a safe gluten threshold for patients with celiac disease. *The American Journal of Clinical Nutrition*.

[118] Ciclitira P. J., Cerio R., Ellis H. J., Maxton D., Nelufer J. M., Macartney J. M. (1985). Evaluation of a gliadin-containing gluten-free product in coeliac patients. *Human Nutrition: Clinical Nutrition*.

[119] Leffler D., Schuppan D., Pallav K. (2013). Kinetics of the histological, serological and symptomatic responses to gluten challenge in adults with coeliac disease. *Gut*.

[120] Anderson R. P., Degano P., Godkin A. J., Jewell D. P., Hill A. V. S. (2000). In vivo antigen challenge in celiac disease identifies a single transglutaminase-modified peptide as the dominant A-gliadin T-cell epitope. *Nature Medicine*.

[121] Tye-Din J. A., Stewart J. A., Dromey J. A. (2010). Comprehensive, quantitative mapping of T cell epitopes in gluten in celiac disease. *Science Translational Medicine*.

[122] Ráki M., Fallang L.-E., Brottveit M. (2007). Tetramer visualization of gut-homing gluten-specific T cells in the peripheral blood of celiac disease patients. *Proceedings of the National Academy of Sciences of the United States of America*.

[123] Brottveit M., Ráki M., Bergseng E. (2011). Assessing possible celiac disease by an HLA-DQ2-gliadin tetramer test. *The American Journal of Gastroenterology*.

[124] Camarca A., Radano G., Di Mase R. (2012). Short wheat challenge is a reproducible in-vivo assay to detect immune response to gluten. *Clinical and Experimental Immunology*.

[125] Hardy M. Y., Tye-Din J. A., Stewart J. A. (2015). Ingestion of oats and barley in patients with celiac disease mobilizes cross-reactive T cells activated by avenin peptides and immuno-dominant hordein peptides. *Journal of Autoimmunity*.

[126] Ontiveros N., Tye-Din J. A., Hardy M. Y., Anderson R. P. (2014). Ex-vivo whole blood secretion of interferon (IFN)-*γ* and IFN-*γ*-inducible protein-10 measured by enzyme-linked immunosorbent assay are as sensitive as IFN-*γ* enzyme-linked immunospot for the detection of gluten-reactive T cells in human leucocyte antigen (HLA)-DQ2·5^+^-associated coeliac disease. *Clinical and Experimental Immunology*.

[127] Tortora R., Russo I., de Palma G. D. (2012). In vitro gliadin challenge: diagnostic accuracy and utility for the difficult diagnosis of celiac disease. *The American Journal of Gastroenterology*.

